# An image classification deep-learning algorithm for shrapnel detection from ultrasound images

**DOI:** 10.1038/s41598-022-12367-2

**Published:** 2022-05-19

**Authors:** Eric J. Snider, Sofia I. Hernandez-Torres, Emily N. Boice

**Affiliations:** Engineering Technology and Automation Combat Casualty Care Research Team, United States Army Institute of Surgical Research, Ft. Sam Houston, TX USA

**Keywords:** Biomedical engineering, Medical research, Ultrasonography

## Abstract

Ultrasound imaging is essential for non-invasively diagnosing injuries where advanced diagnostics may not be possible. However, image interpretation remains a challenge as proper expertise may not be available. In response, artificial intelligence algorithms are being investigated to automate image analysis and diagnosis. Here, we highlight an image classification convolutional neural network for detecting shrapnel in ultrasound images. As an initial application, different shrapnel types and sizes were embedded first in a tissue mimicking phantom and then in swine thigh tissue. The algorithm architecture was optimized stepwise by minimizing validation loss and maximizing F1 score. The final algorithm design trained on tissue phantom image sets had an F1 score of 0.95 and an area under the ROC curve of 0.95. It maintained higher than a 90% accuracy for each of 8 shrapnel types. When trained only on swine image sets, the optimized algorithm format had even higher metrics: F1 and area under the ROC curve of 0.99. Overall, the algorithm developed resulted in strong classification accuracy for both the tissue phantom and animal tissue. This framework can be applied to other trauma relevant imaging applications such as internal bleeding to further simplify trauma medicine when resources and image interpretation are scarce.

## Introduction

Currently, ultrasound (US) imaging is commonly used for both detecting foreign bodies during emergency medicine and also assessing internal injuries and traumas faced in remote environments due to its high accuracy, portability, and modest power requirements^1–3^. While higher fidelity imaging modalities are preferred in the hospital setting, this is often not possible in remote settings, such as with combat casualty care. In fact, the Extended Focused Assessment with Sonography for Trauma (eFAST) examination protocol is the current standard of care on the battlefield when internal injuries are suspected^4^. In addition, US imaging can be valuable after improvised explosive devices (IED) and other blast related injuries have occurred to detect any foreign bodies (i.e., shrapnel) embedded in tissue that may cause infections^5^ or be responsible for internal hemorrhage^6^.

However, an ultrasound exam is only effective if a trained sonographer procures clear US images in the correct locations and accurately interprets them. Unfortunately, the utilization of US devices for diagnosing critical internal injuries is technically challenging with varying degrees of injury and patient position^7^, requiring hours of end-user training^8^, and is normally done by a skilled ultrasonography technician. As a result, there is a clear need to simplify the US image acquisition and evaluation process so that casualties can be properly assessed for internal injuries on the battlefield or remote environments by non-expert providers with limited training.

Ultrasound image interpretation algorithms have been developed for a wide variety of applications, from tumor detection^9^, to thyroid nodules^10^, and lung pathologies due to COVID-19^11^. Image classification algorithms such as these primarily rely on supervised deep learning convolutional neural networks (CNN) to detect trends between positive and negative image sets. Higher fidelity algorithms can move beyond simple classification into object detection or segmentation approaches to highlight the abnormality in the tissue^12–14^ or utilize US video footage instead of image sets^15–17^.

Oftentimes, these algorithms are built via transfer learning to existing algorithm architectures for additional applications, such as the existing VGG-16^18–20^, EfficientNet^21–23^, and InceptionNet^24–26^ architectures trained on millions of image sets from the ImageNet archive^27^. However, for such robust performance across 1000s of image classification types, these algorithms can be cumbersome and overly complicated when fewer categorical image classification tasks are desired. This is especially critical if the ultimate goal is to integrate these algorithms within existing US imaging systems for near real-time diagnoses. Here, we develop and optimize a streamlined algorithm specifically for shrapnel detection in ultrasound images, a key step towards developing algorithms for automating military or remote medicine relevant US diagnoses.

## Methods

### Ultrasound image acquisition

Ultrasound images were collected from gelatin phantom or swine tissue using a Sonosite Edge (Fujifilm, Bothell, WA, USA) and HF50 probe (Fujifilm, Bothell, WA, USA). Phantoms were tuned for mimicking tissue properties^28^ and were comprised of a plastic bone, inner muscle layer, and outer fat mimicking layer. The inner layer base was 10% (w/v) gelatin with 0.25% (w/v) flour in 2:1 (v/v) water to evaporated milk, containing 2% agarose (w/v) solid components with various flour amounts in the agarose to heterogeneously adjust the echogenicity. The outer fat-mimicking layer was the same base 10% gelatin solution with 0.10% (w/v) flour. Baseline images were captured at varying depths and with longitudinal and transverse probe placement before shrapnel insertion. An average of 15 images was taken in each quadrant of the phantom, approximately every 90º. After baseline images were collected, we progressively inserted the shrapnel fragments (Table 1A) using surgical forceps. An average of 12 images for each of the 7 shrapnel types, per thigh model were collected. Shrapnel depth within the phantom varied from 2 cm up to 5.5 cm, where it was almost impacting the bone. Due to material properties of gelatin, the shrapnel slid easily into the muscle layer of the phantom. After two or three shrapnel pieces had been inserted in the same location, we rotated the thigh between 60–90º, or until the US trace of the previous insertion was out of view. In total, we collected baseline and shrapnel positive US images from 14 phantom thigh models.

**Table 1:** Overview of shrapnel types used and total number of images for training, validation, and testing.

(A)	Object type		Minimum width (mm)		Maximum width (mm)	
	Metal		5		16	
	Ceramic		9		10	
	Plastic		7		15	
	Asphalt		4		8	
	Gravel		5		8	
	Glass		2		11	
	Rubber		5		15	
	Wood		8		20	

(B)	Phantom image numbers during model optimization		Phantom image numbers for final model		Swine image numbers for final model	
	Baseline	Shrapnel	Baseline	Shrapnel	Baseline	Shrapnel
Total number of images	954	1132	606	720	554	584
Model training split	611	724	332	395	355	374
Training validation split	152	181	83	98	88	93
Model testing split	191	227	191	227	111	117

(A) Shrapnel were irregularly shaped, so the approximate longest and shortest width of the objects are reported. All objects were hyperechoic relative to the phantom or swine tissue. (B) Images were randomly distributed between each group for both baseline and shrapnel image classification. The process was repeated for all phantom images, all swine images, and a subset of the phantom images to be more similar in number to the swine image sets for consistency during final model evaluation.

A similar methodology was followed for collecting baseline and shrapnel images from recently euthanized swine thighs. All experiments were performed in accordance with a protocol approved by the local Institutional Animal Care and Use Committee at the US Army Institute of Surgical Research. All methods were carried out in accordance with relevant guidelines and regulations. The authors also complied with the ARRIVE (Animal Research: Reporting of In Vivo Experiments) guidelines. Baseline images of the femoral bicep muscle of three euthanized porcine subjects were acquired with the same ultrasound setup. An average of 150 images came from each animal using a combination of video clips and still images. The overall collection of images had varying depths, angles, and bone appearances similar to gelatin phantom image acquisition. Two or three small incisions were made on different locations of the euthanized swine’s bicep and the same shrapnel types were inserted. An average of 30 images was taken for every shrapnel type changing location of the shrapnel and angles of the ultrasound probe. Depths of shrapnel pieces varied from 2 cm up to 4.5 cm into the thigh tissue. All the images for both, swine and phantom, were compiled for further image preprocessing.

### Image preprocessing

Ultrasound images were divided into four groups: (i) phantom baseline, (ii) phantom shrapnel, (iii) swine baseline, and (iv) swine shrapnel. To increase the number of images prior to data augmentation and model training, a custom script was used to extract the frames from acquired ultrasound videos using the ffmpeg-Ruby tool (version 4.4). Next, all images were cropped to a square aspect ratio to remove the settings, file name, and other information included in exported ultrasound images or video frames (Supplemental Fig. 1). The images were then converted to 16bit and resized to 512 × 512 pixels. All preprocessing was performed by batch processing in FIJI^29,30^.

The four groups of preprocessed images were then randomly split into 80% training/validation and 20% testing sets using Python 3.8 (split-folder 0.4.3 package). The testing set of images was only used for backend testing of an optimized model and remained isolated during training. The remaining images were used for training and iterative validation during the training process. Within the image classification algorithm, the training set was further randomly split into 80% of the images being used for training and the remaining 20% for iterative model validation each epoch. As there were more gelatin phantom images than swine images, when comparing the algorithm performance between each image type, the phantom image set numbers were reduced to be closer in number to the swine image sets (Table 1B). However, throughout initial model optimization (section “Algorithm Stepwise Optimization”), the entire phantom image set was used exclusively. Test image sets were used for backend external testing of each model after training using both phantom and swine image sets, as well as phantom test images subdivided into the 8 shrapnel types.

### Algorithm design

**Figure 1:**
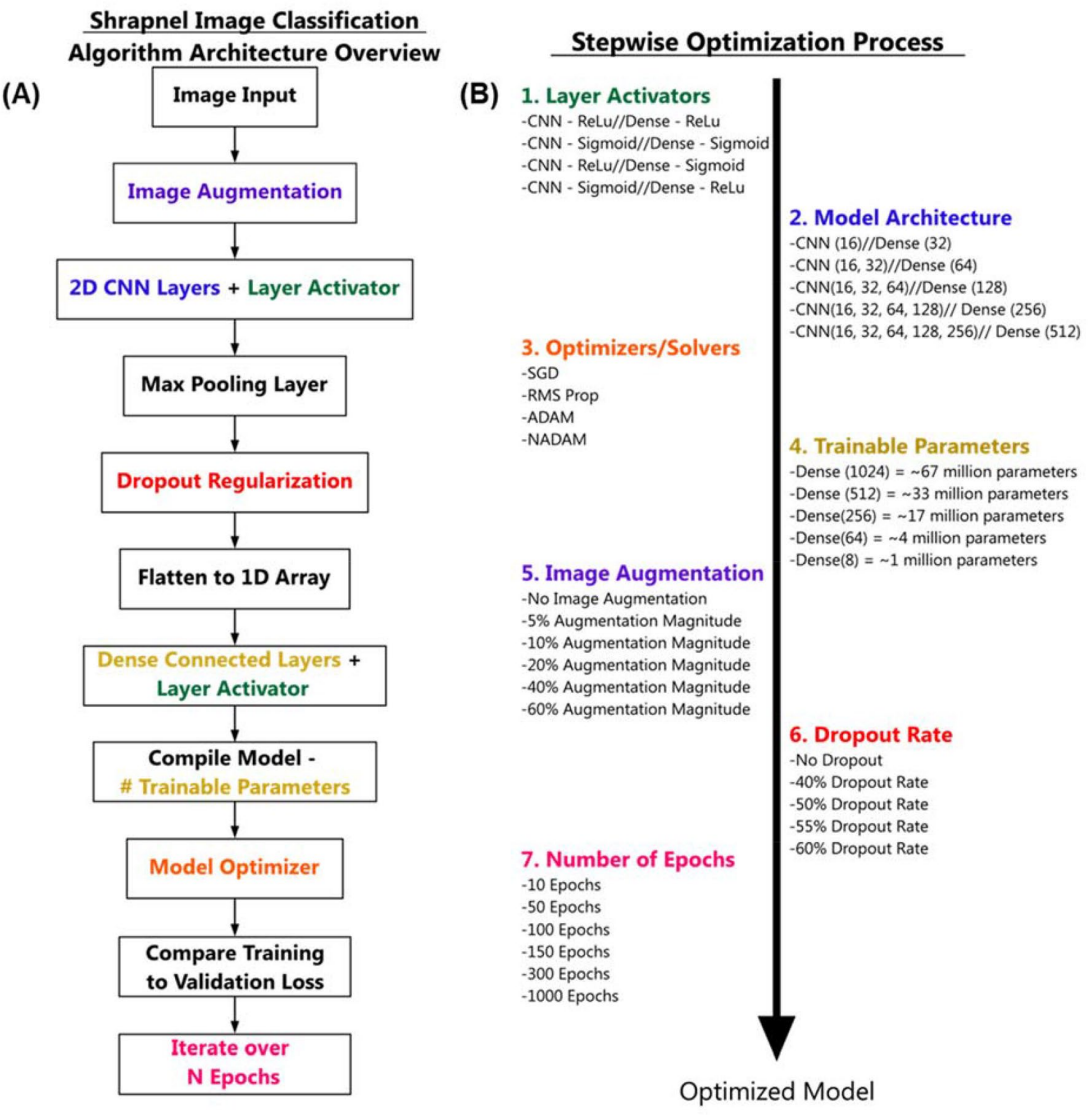
Overview of the Shrapnel Image Classification Framework and Stepwise Optimization Process. (**A**) Process diagram for the image classification algorithm. Images were imported and data augmentation was applied prior to feature identification through the 2D CNN layers and corresponding layer activators. Each CNN layer was followed by a max pooling layer. After 2D CNN layers, a dropout regularization layer was added to mitigate training overfitting. Images were then flattened, and densely connected layers were included until only two categorical outcomes could be selected: shrapnel or baseline. The model was compiled for a summation of the trainable parameters, followed by training optimization by minimizing loss through each epoch iteration. (**B**) Summary of the stepwise features evaluated during model evaluation. Each step is color-coded to its position in the model framework and the parameter variables evaluated are shown.

The deep learning image classification algorithm was designed using TensorFlow/Keras (version 2.6.0) in Python (version 3.8) using Jupyter Notebook. The TensorFlow framework for importing images, loading the neural network architecture, and displaying the results followed readily available resources from TensorFlow^31^ (Fig. 1). Briefly, images were imported, and the validation and training splits were generated followed by verification that the proper images were loaded using Matplotlib. The initial architecture of the algorithm was designed to include image augmentation and dropout regularization similar to previous research efforts to minimize overfitting^32^ (Fig. 1). The framework for the algorithm applied image augmentation through flip, rotation, zoom, and contrast adjustment transformations at specified magnitudes. Next, 2D sparsely connected convolutional neural network layers and layer activators were arranged with 2D max pooling layers. Different CNN architectures were evaluated (see “Algorithm Stepwise Optimization”), but after the CNN layers, a dropout regularization layer was included with an initial rate of 50%, followed by a flatten layer to reduce the images to a 1D array. Then, 2 densely connected layers were included with the final layer rendering only 2 possible outcomes: shrapnel or baseline. Initially, 100 epochs were run using ADAM as the optimizer minimizing loss. Validation and training loss were used to optimize the model through the iteration and plotted to track overall model convergence after training was complete.

To optimize the overall model architecture specifically for shrapnel ultrasound image classification, different layers and attributes of the algorithm were modified stepwise to determine its effect on overall performance. Overall performance was determined by the magnitude of the validation loss averaged over the final five epochs and the F1 score from model predictions for test phantom image sets. Seven model features were optimized in succession: (1) layer activators, (2) model architecture, (3) optimizers/solvers, (4) trainable parameters, (5) image augmentation, (6) dropout rate, and (7) number of epochs (Fig. 1); where each parameter was modified 4 to 6 ways as shown. One model version was selected before moving onto the next variable until each parameter was tuned for shrapnel ultrasound image classification. The final, optimized algorithm architecture used is provided in Supplementary File 2.

### Performance metrics

Model prediction in test image sets was performed after the model was trained with a separate Python script which loaded the trained model and performed independent predictions on every image within the test sets. Class prediction, true category, and prediction confidence were exported for each image in the test data set. These results were used to calculate overall trained model performance metrics: accuracy, precision, recall, specificity, and F1 score. Receiver Operating Characteristic (ROC) Curves, area under the ROC curve (AUC), Confusion Matrices, and Calibration Plots were generated using GraphPad Prism 9 (San Diego, California, USA) by exporting the predictions and confidences for the entire set of test images. This process was repeated for both phantom and swine test image sets. Lastly, phantom shrapnel type results were evaluated for overall accuracy of prediction and prediction confidence for the eight shrapnel types. After the optimal model was identified, three replicate models were trained and tested as described above, using different training-validation split seeds to introduce training variability and test the robustness of the model’s performance.

## Results

### Stepwise optimization for ultrasound image classification

As detailed in Fig. 1, we optimized the shrapnel image classification model across seven criteria based on current trends^33^: (1) layer activators, (2) overall CNN architecture, (3) model optimizers, (4) number of trainable parameters, (5) image augmentation, (6) dropout regularization, and (7) number of epochs. First, two-layer activation functions, rectified linear activation function (ReLu) or Sigmoid, were evaluated for the 2D CNN layers and 1D dense architecture layers. ReLu and sigmoid activation functions were selected based on their utility in various network architectures, such as AlexNet and EfficientNet. Overall, ReLu was selected for the 2D CNN layers and sigmoid was selected for the 1D dense, fully connected layer (Table 2A). This combination performed slightly better compared to when ReLu was used for the dense layers, with a high F1 score, a low validation loss compared to the other layer activator configurations. Second, the overall CNN layer architecture was modified but the total trainable parameters were fixed at approximately 33 million by keeping the dense layer 2 × the size of the final CNN layer. Overall, the higher layer number CNN architectures performed better compared to the fewer layer versions (Table 2B). Performance was slightly improved at the highest layer depth architecture evaluated which was selected for future optimization. This architecture, while it did not have the lowest validation loss, had the best F1 score at 0.96.

**Table 2:** Summary of performance metrics for stepwise optimization steps.

**(A)** CNN	Dense	Validation loss	F1 score	Accuracy	AUC
ReLu	ReLu	0.38	0.94	0.94	0.93
Sigmoid	Sigmoid	0.69	0.70	0.54	0.50
**ReLu**	**Sigmoid**	**0.29**	**0.93**	**0.93**	**0.93**
Sigmoid	ReLu	0.36	0.90	0.88	0.87

**(B)**CNN architecture	Dense nodes	Validation loss	F1 score	Accuracy	AUC
16	32	0.69	0.70	0.54	0.50
16, 32	64	0.69	0.70	0.54	0.50
16, 32, 64	128	0.29	0.93	0.93	0.93
16, 32, 64, 128	256	0.23	0.94	0.94	0.94
**16, 32, 64, 128, 256**	**512**	**0.29**	**0.96**	**0.95**	**0.95**

(C) Optimizer/solver		Validation loss	F1 score	Accuracy	AUC
SGD		0.30	0.94	0.93	0.93
**RMS Prop**		**0.17**	**0.93**	**0.93**	**0.93**
ADAM		0.29	0.96	0.95	0.95
NADAM		0.28	0.95	0.95	0.95

**(D)** Dense nodes	Trainable parameters	Validation loss	F1 score	Accuracy	AUC
1024	67,504,546	0.18	0.96	0.96	0.96
512	33,948,578	0.17	0.93	0.93	0.93
**256**	**17,170,594**	**0.16**	**0.96**	**0.96**	**0.96**
64	4,587,106	0.17	0.95	0.94	0.94
8	916,922	0.23	0.95	0.95	0.94

Average validation loss for the final 5 epochs during model training and F1 scores calculated from model predictions on gelatin phantom image sets are shown for four model optimization parameters: (A) Layer activators for the 2D CNN and 1D Dense neural network layers, (B) 2D CNN layer architecture, (C) model optimizer, and (D) total number of trainable parameters. Bold text font in each table region represents the selected model architecture that was used for subsequent optimization steps.

Third, a total of four different model compilation optimizers were compared from established models, such as InceptionV3, EfficientNet, VGG-16, and ResNet^34,35^: (a) stochastic gradient descent (SGD), (b) RMS Prop, (c) ADAM, (d) NADAM. Performance was similar across each with the exception of the RMS prop optimizer which resulted in the greatest reduction in validation loss compared to the others and was thus selected as the optimizer for future runs (Table 2C). Fourth, the number of trainable parameters was modified by adjusting the dense, fully-connected layer architecture size. Overall, parameter number did not impact model performance greatly, with approximately 1 million parameters increasing validation loss minimally (Table 2D). However, the approximately 17 million parameter setup had slightly improved F1 and validation loss scores and was selected as the preferred model architecture.

**Figure 2:**
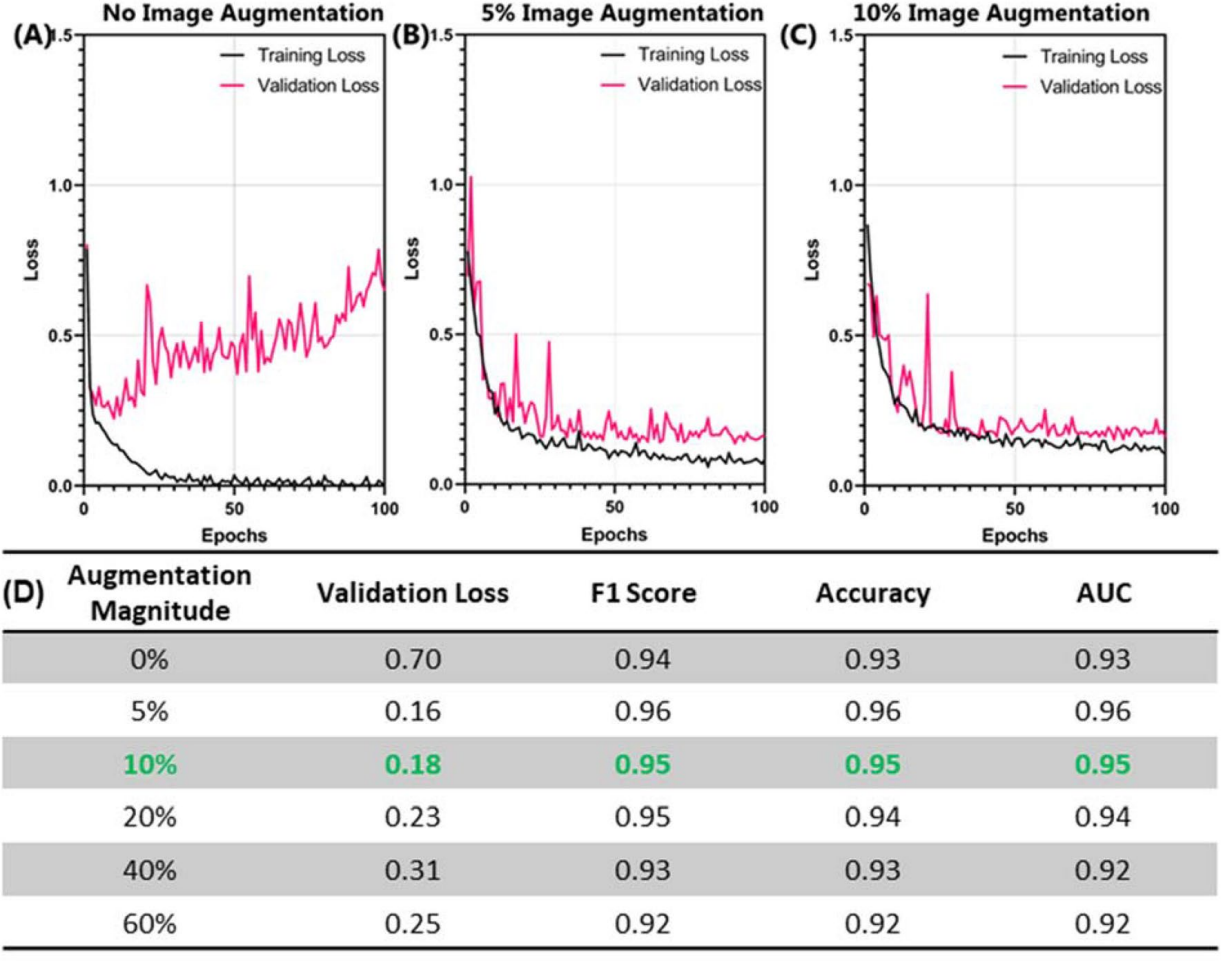
Effect of image augmentation on model performance. (**A–C**) Training and Validation loss plots for models with (**A**) no image augmentation, (**B**) 5% image augmentation and (**C**) 10% image augmentation for the 100 epochs. (**D**) Summary of performance statistics for the different magnitudes of the image augmentation. Performance is shown for average validation loss for the last 5 epochs and F1 score from the test phantom images. Green text font represents the selected model architecture for subsequent optimization steps.

Next, we investigated the impact of image augmentation on model performance. Image augmentation was introduced 4 ways: (1) image flip, (2) zoom, (3) rotation, and (4) contrast. When no augmentation was introduced the validation loss was much higher (0.70), which is further reflected by the training and validation loss plot across the epochs, indicating overfitting of the model (Fig. 2A). Overfitting was greatly reduced for all augmentation magnitudes evaluated (Fig. 2B–D). Based on the model’s F1 score and similarity between training and validation loss (Fig. 2), 10% augmentation was selected as the optimal augmentation magnitude to prevent overfitting while eliminating the introduction of unnecessary noise (Fig. 2C).

**Figure 3:**
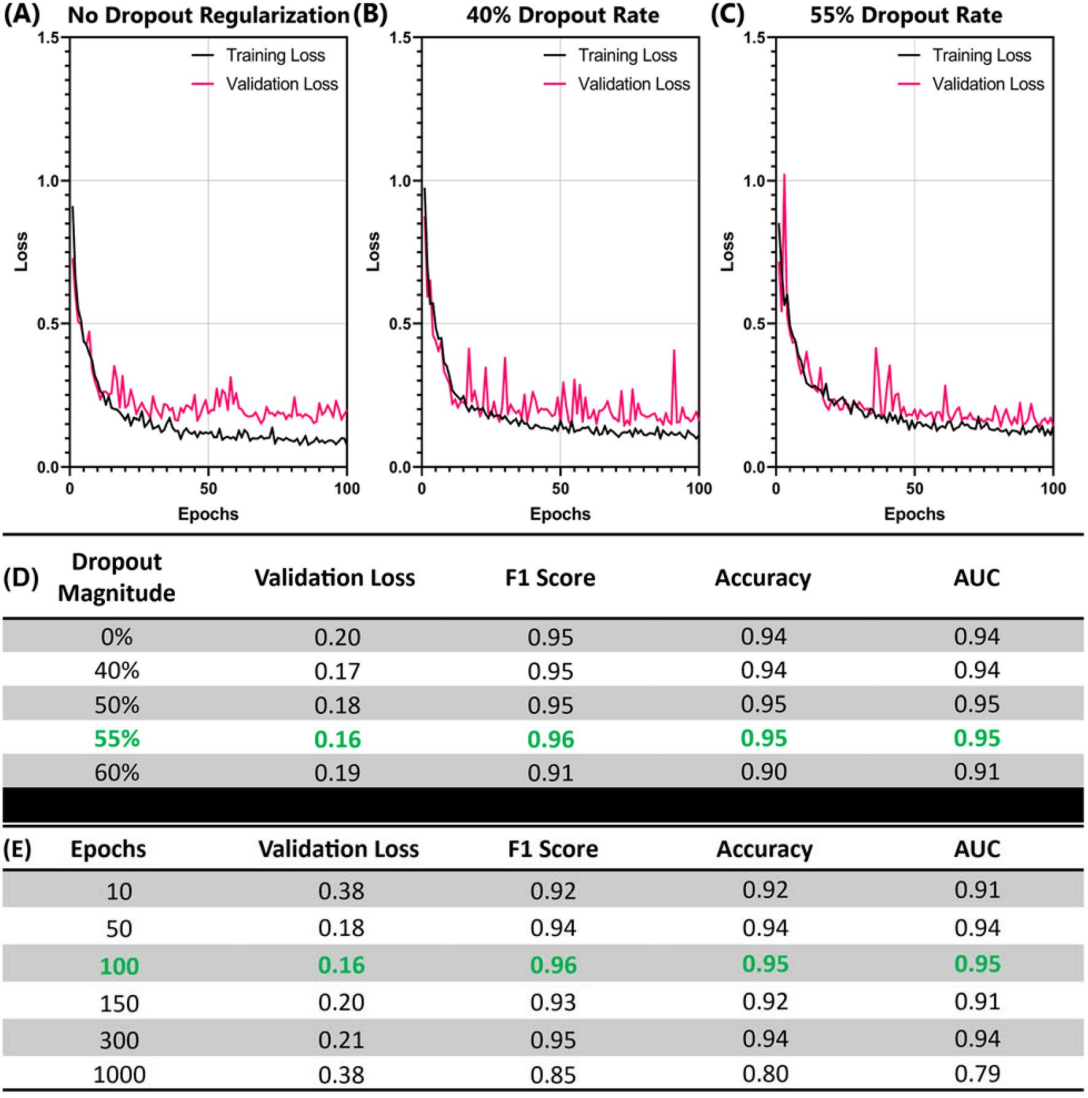
Effect of dropout regularization and number of epochs on model performance. (**A–C**) Training and validation loss plots for models with (**A**) no dropout regularization, (**B**) 40% dropout and (**C**) 55% dropout for the 100 epochs. (**D, E**) Summary of performance statistics for the different magnitudes of (**D**) dropout regularization and (**E**) number of epochs. Performance is shown for average validation loss for the last 5 epochs and F1 score from the test phantom images. Green text font represents the selected model architecture for subsequent optimization steps.

An additional approach to reduce overfitting is dropout regularization and a dropout rate of 50% was selected as an ideal starting point similar to rates used in AlexNet. Different dropout rates were evaluated from none to up to 60% after the final 2D CNN layer in the model. Overall, the dropout regularization magnitude on top of the image augmentation already set at 10% had a subtle effect, but improved performance was evident when dropout was removed (Fig. 3). The 55% rate was selected as it improved each critical parameter slightly over the 50 and 60% dropout rate scenarios (Fig. 3D). Lastly, the number of epochs during training was modulated to determine its impact on model convergence. As shown from the performance metrics, too few epochs resulted in worse performance, but 50 epochs were comparable to 100 epochs. Increasing the number of epochs started to show divergence of the validation and training results and resulted in poorer performance (Fig. 3E). As a result, 100 epochs were kept as the optimal hyperparameter selection. The final model architecture after each stepwise optimization is shown in Supplementary Fig. 2.

### Performance of the optimized shrapnel classification algorithm

**Figure 4:**
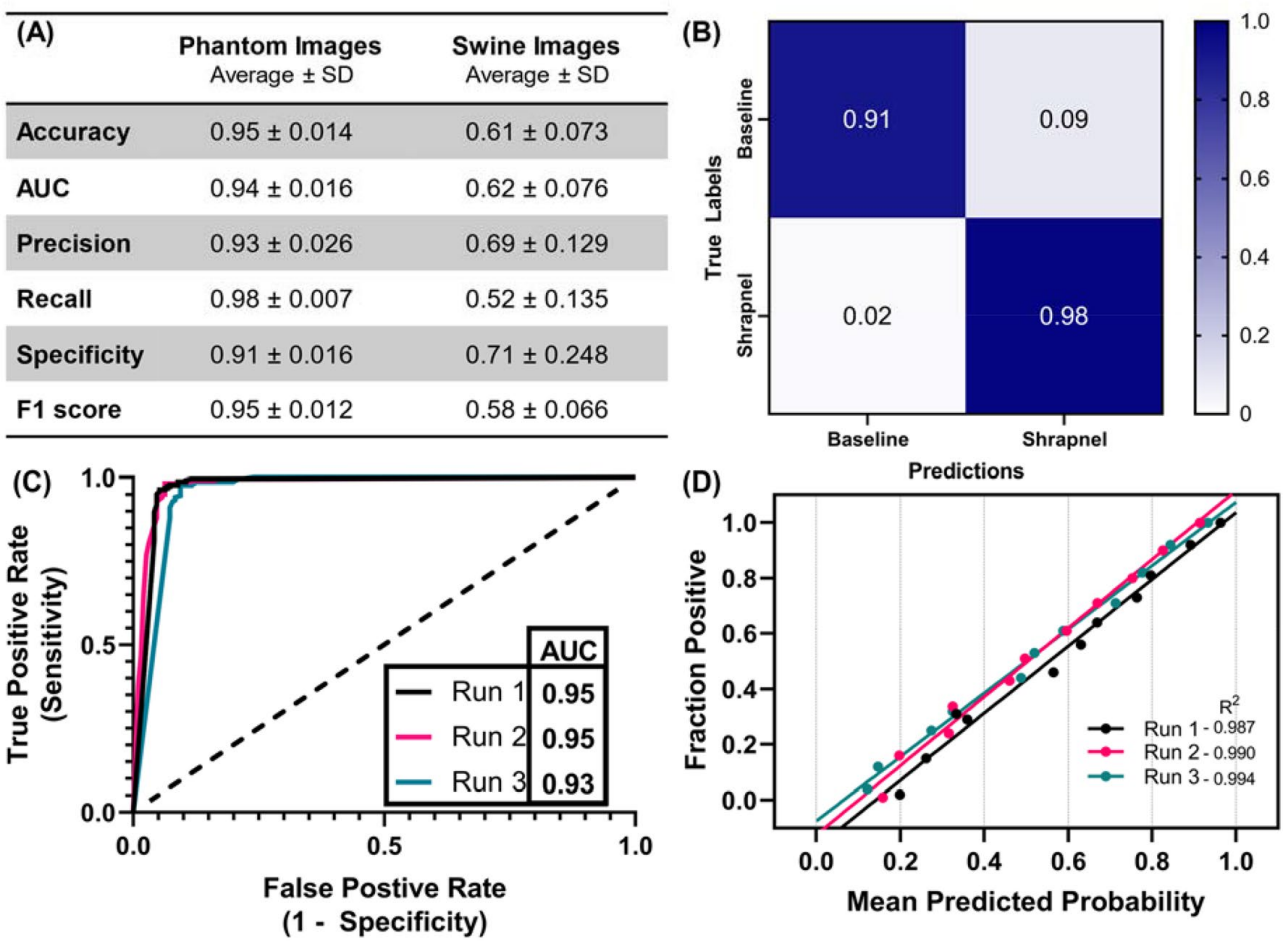
Performance of the Optimized Model using Phantom Image Training. Three replicate models were created with different randomized training-validation splits. (**A**) Tabular statistics for phantom images and swine images. Average and standard deviation (SD) are shown for each statistic. Note that the shown swine predictions are from a model trained only on a phantom image set. (**B**) Average confusion matrix performance. (**C**) ROC curve and (**D**) calibration plots for test data sets.

After stepwise model optimization, we evaluated the selected model’s performance across several key criteria and using three different training-validation random splits to modify training. Using the phantom image sets to train the model, overall classification accuracy was approximately 95% among phantom test image sets. Precision and recall were approximately 93% and 98% respectively, indicating a slightly higher false positive outcome than false negative outcome, which was reflected in the confusion matrix and the lower 91% specificity score (Fig. 4A,B). The ROC curve resulted in an area under the curve value of approximately 0.94 (Fig. 4C). In addition, a calibration plot was constructed by binning the images into different shrapnel (positive) image fractions and calculating the average prediction confidence for these various fractions, with a well-trained model having similar prediction confidence to each fractional image set. Overall, the predictions had a linear correlation to the fractional splits, but there was an offset in performance between the three replicate models (Fig. 4D).

**Figure 5:**
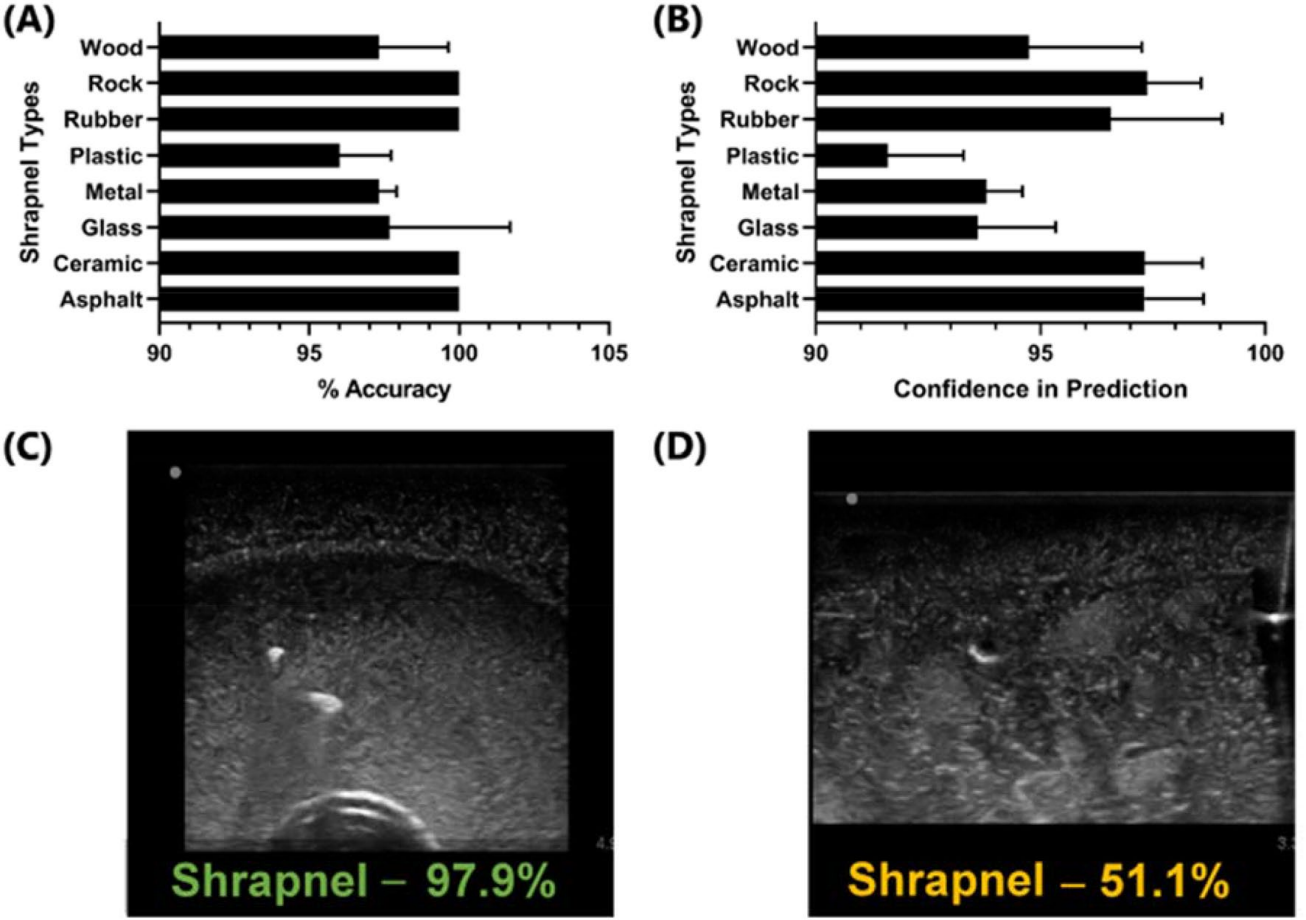
Model Performance for Different Shrapnel Types. (**A**) Accuracy and (**B**) prediction confidence for test phantom image sets for eight different shrapnel types: Asphalt (n = 33), Ceramic (n = 28), Glass (n = 14), Metal (n = 59), Plastic (n = 34), Rubber (n = 10), Rock (n = 29), Wood (n = 23). Results are shown as average values, and errors bars denote standard deviation. Representative images for glass shrapnel type for (**C**) high prediction confidence and (**D**) low prediction confidence. Additional representative images for all other shrapnel types are provided in Supplementary Fig. 3.

We also separated the shrapnel into 8 types and highlighted the accuracy and prediction confidence for each. Overall, all types had high prediction accuracy, but there were differences between the types, with ceramic performing well while smaller less echogenic types such as wood were harder to detect (Fig. 5). Representative images for each shrapnel type are shown in Supplementary Fig. 3 to highlight the prediction and image variability for each. Lastly, the phantom trained model was also used to blindly evaluate swine ultrasound images, to determine how closely the phantom training approach can be applied to swine image sets without transfer learning to this new image type. Performance was poor, with overall accuracy at 61% with an F1 score of 0.58 (Fig. 4A).

### Performance for swine shrapnel detection using porcine image sets

**Figure 6:**
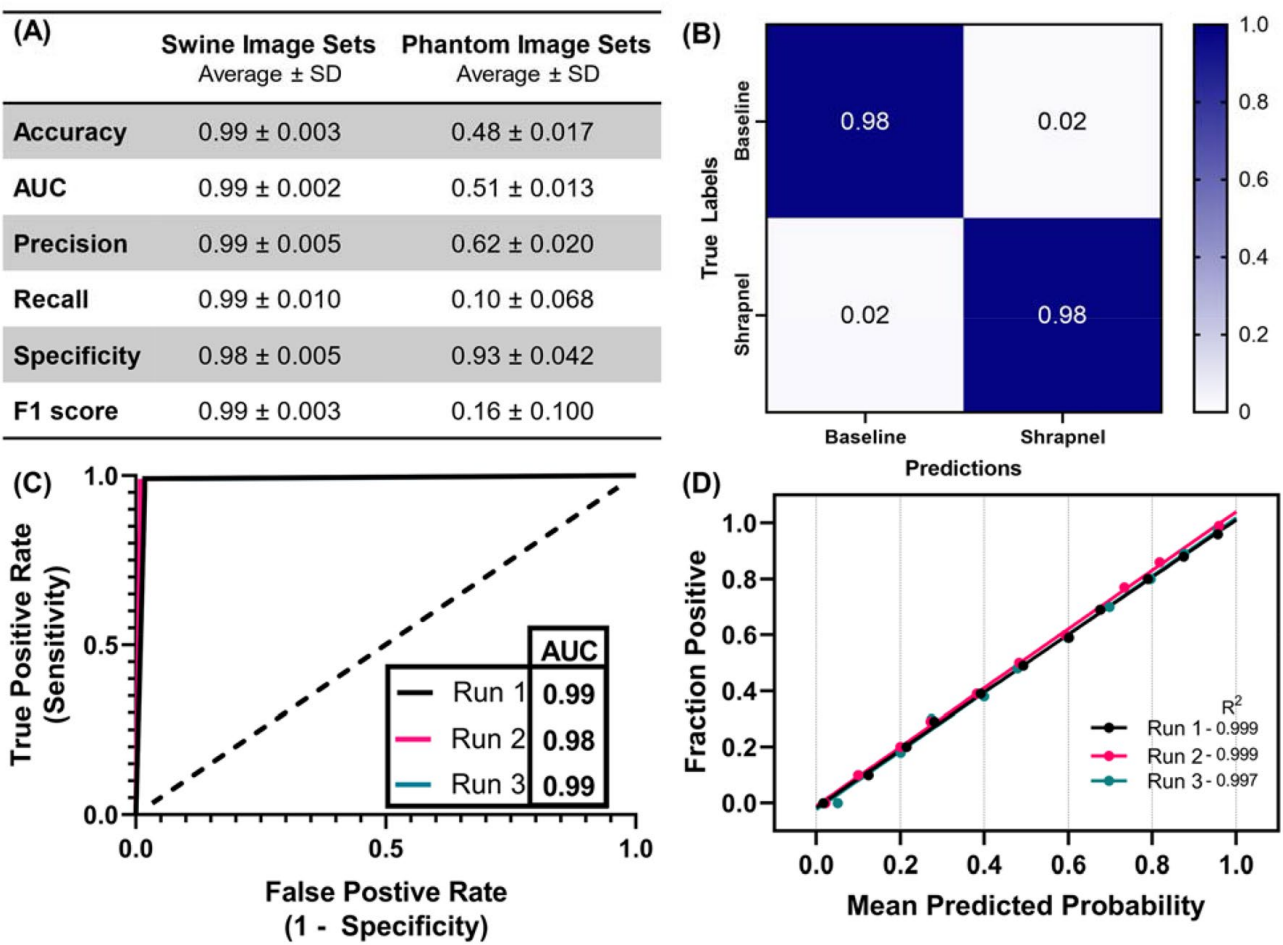
Performance of the Stepwise Optimized Model using Swine Image Training. Three replicate models were created with different randomized training-validation splits. (**A**) Tabular statistics for swine images and phantom images. Average and standard deviation (SD) are shown for each statistic. Note that the shown phantom predictions are from a model trained only on a swine image set. (**B**) Average confusion matrix performance. (**C**) ROC curve and (**D**) calibration plots for swine test data sets.

Next, the model was retrained using entirely different image data sets collected from swine instead of phantom. Swine testing image set performance reached as high as 99% accuracy with all performance metrics having a score of 0.98 or better (Fig. 6A). False detection rates remained low as shown by confusion matrix and the ROC-AUC reached 0.99 as well (Fig. 6B,C). The calibration plot had a strong linear correlation with less noise between the various replicate trained models compared to the shrapnel trained model (Fig. 6D). Overall, the model performed well for the swine image sets, performing better than the model performed when trained on phantom image sets alone. As expected, however, the accuracy to detect shrapnel in the phantom image sets was drastically reduced in this scenario with accuracy reduced to 48% (Fig. 6A). Similar poor scores were evident for all other performance metrics, with a recall and F1 score being the lowest scoring metrics. This is analogous to poor model performance for swine images when the model was exclusively trained on phantom images.

## Discussion

Ultrasound imaging is essential for medical diagnostics in emergency medicine and combat casualty care. Unfortunately, image interpretation requires specialized expertise that often is not available in remote environments, limiting the utility for medical imaging for critical triage decisions. As a result, lowering the expertise threshold is critical for making medical imaging diagnosis accessible in these scenarios. Here, we describe a deep-learning algorithm for this purpose. Specifically, the image classification model developed here is optimized for shrapnel detection in phantom or animal tissue.

To highlight the effect of various parameters on model performance, we optimized each aspect of the model in a stepwise manner. In total, seven categories were tuned, such as number of trainable parameters, and layer activators, as well as image augmentation and dropout regularization to combat model overfitting. While there are many additional factors that can be optimized and more refined methods such as Bayesian hyperparameter optimization are possible^36,37^, the approach taken helped highlight the effect of each parameter on overall model performance. For instance, insufficient CNN layers resulted in poor performance as did certain layer activation configurations. With the finite number of images used with this model, training was susceptible to overfitting when the image augmentation was removed. Overfitting was evident as training loss approached zero while loss calculations during validation did not converge and remained high as the model was fit for the specific images in training set only. With the benefit of image augmentation, dropout regularization’s effect was not as evident, but its effect may have been more evident without image augmentation. A more robust data augmentation methodology such as mixup^38–40^ can be utilized to further reduce overfitting. Overall, this manual stepwise model optimization approach was able to decide on a model tuned to this specific imaging application.

With the model optimized for this application, we highlighted its performance for shrapnel detection for both phantom and swine image sets. Optimization was conducted on phantom image sets as they could be acquired more easily and in greater number than swine images. The final model reached approximately 95% accuracy for test image sets. This was a stronger performance metric than we anticipated in the phantom images as they had variable compositions across some of the gelatin phantoms used and far fewer images were used in training the model compared to other ultrasound imaging applications^41,42^. In general, the model had higher false negative rates than false positives rates, indicating shrapnel was more likely to be undetected by the algorithm than a baseline being incorrectly categorized. From a medical diagnostic perspective, it likely would be preferred to have a higher positive rate to minimize shrapnel embedded in tissue going unnoticed, so a prediction confidence threshold below 50% may be needed to produce this result. A range of shrapnel sizes, materials, and shapes were evaluated, and performance was similar for most types, indicating the false negative rate was less influenced by the shrapnel type and may have been influence by other factors. Interestingly, when the model was trained on swine image sets, accuracy for swine images was nearly 99% and the false negative rate was reduced. The phantom designed for this application had more iterations and independent replicates while the swine image sets came predominately from US video frames taken from only three animal thighs. This lower image diversity may explain the performance differences, but overfitting was not detected with training and validation loss parameters.

Future work can proceed various ways. First, model performance could be further tuned by utilizing Bayesian hyperparameter optimization with the ratio image sets as the training data^36,37^. This optimization approach can tune multiple parameters and logically decide which pairings best can minimize loss or other performance metrics. Second, the model type can be reconfigured for object detection to identify the shrapnel location in the image^17^. This may be ideal for further diagnostic purposes and could be used for assisting during surgical interventions. Third, the model can be retrained for other injury conditions and anatomical locations relevant to austere medicine or combat casualty care. Shrapnel detection is more essential in the abdomen where internal bleeding may occur and in the eyes where intraocular foreign bodies and open globe injuries can lead to loss of vision^43–45^. Fourth, other image interpretation applications such as for pneumothorax, hemothorax, and abdominal hemorrhage will be explored to automate eFAST examination diagnosis for military and emergency medicine^8^. Fifth, future work will compare overall model performance to configurations of widely used networks such as InceptionNet^46^ or EfficientNet^47^ to highlight strengths or weaknesses of the developed model and to confirm that its performance and overall processing speed compare favorably to other models. In conclusion, the algorithm developed in this work was optimized for high performance detection for identifying shrapnel in both phantom and swine image sets, an important step in simplifying ultrasound image diagnosis on the battlefield and trauma situations.

## Supplementary Information

Supplementary Information.

## Data Availability

The datasets generated during and/or analyzed during the current study are available from the corresponding author on reasonable request.

## References

[1] Radwan MM, Abu-Zidan FM (2006). Focussed assessment sonograph trauma (FAST) and CT scan in blunt abdominal trauma: Surgeon’s perspective. Afr. Health Sci..

[2] Scalea TM, Rodriguez A, Chiu WC, Brenneman FD, Fallon WF, Kato K (1999). Focused assessment with sonography for trauma (FAST): Results from an international consensus conference. J. Trauma..

[3] Hill R, Conron R, Greissinger P, Heller M (1997). Ultrasound for the detection of foreign bodies in human tissue. Ann. Emerg. Med..

[4] Harper, H., Myers, M. Military and tactical ultrasound. Emergency Ultrasound (2008)

[5] Wolf JM, Bucknell A (2010). Arthroscopic removal of improvised explosive device (IED) debris from the wrist: A case report. Mil. Med..

[6] Shuker ST (2012). The immediate lifesaving management of maxillofacial, life-threatening haemorrhages due to IED and/or shrapnel injuries: “when hazard is in hesitation, not in the action”. J. Craniomaxillofac. Surg..

[7] Bloom BA, Gibbons RC. Focused Assessment with Sonography for Trauma. In: StatPearls [Internet]. Treasure Island (FL): StatPearls Publishing; 2021 [cited 2021 Nov 30]. Available from: http://www.ncbi.nlm.nih.gov/books/NBK470479/. 29261902

[8] Pencil K (2017). eFAST simulation training for trauma providers. J. Trauma Nurs..

[9] Chiang T-C, Huang Y-S, Chen R-T, Huang C-S, Chang R-F (2019). Tumor detection in automated breast ultrasound using 3-D CNN and prioritized candidate aggregation. IEEE Trans. Med. Imaging..

[10] Yu X, Wang H, Ma L (2020). Detection of thyroid nodules with ultrasound images based on deep learning. Curr. Med. Imaging Rev..

[11] Born J, Wiedemann N, Cossio M, Buhre C, Brändle G, Leidermann K (2021). Accelerating detection of lung pathologies with explainable ultrasound image analysis. Appl. Sci..

[12] Li H, Weng J, Shi Y, Gu W, Mao Y, Wang Y (2018). An improved deep learning approach for detection of thyroid papillary cancer in ultrasound images. Sci. Rep..

[13] Baumgartner CF, Kamnitsas K, Matthew J, Fletcher TP, Smith S, Koch LM (2017). SonoNet: Real-time detection and localisation of fetal standard scan planes in freehand ultrasound. IEEE Trans. Med. Imaging.

[14] Vakanski A, Xian M, Freer PE (2020). Attention-enriched deep learning model for breast tumor segmentation in ultrasound images. Ultrasound Med. Biol..

[15] Xu Q, Hamilton RJ (2006). A novel respiratory detection method based on automated analysis of ultrasound diaphragm video. Med. Phys..

[16] Gemignani V, Faita F, Ghiadoni L, Poggianti E, Demi M (2007). A system for real-time measurement of the brachial artery diameter in B-mode ultrasound images. IEEE Trans. Med. Imaging.

[17] Wu X, Tan G, Zhu N, Chen Z, Yang Y, Wen H (2021). CacheTrack-YOLO: Real-time detection and tracking for thyroid nodules and surrounding tissues in ultrasound videos. IEEE J. Biomed. Health Inform..

[18] Tammina S (2019). Transfer learning using VGG-16 with deep convolutional neural network for classifying images. Int. J. Sci. Res. Publ. (IJSRP)..

[19] Wu, Y., Qin, X., Pan, Y., Yuan, C. Convolution Neural Network based Transfer Learning for Classification of Flowers. In: 2018 IEEE 3rd International Conference on Signal and Image Processing (ICSIP). 2018. p. 562–6.

[20] Shi Z, Hao H, Zhao M, Feng Y, He L, Wang Y (2019). A deep CNN based transfer learning method for false positive reduction. Multimed. Tools Appl..

[21] Miglani V, Bhatia M, Hassanien AE, Bhatnagar R, Darwish A (2021). Skin lesion classification: a transfer learning approach using efficientnets. Advanced Machine Learning Technologies and Applications.

[22] Khobragade V, Jain N, Sisodia DS, Florez H, Misra S (2020). Deep transfer learning model for automated screening of cervical cancer cells using multi-cell images. Applied Informatics.

[23] Munien C, Viriri S (2021). Classification of hematoxylin and eosin-stained breast cancer histology microscopy images using transfer learning with efficientnets. Comput. Intell. Neurosci..

[24] Buddhavarapu VG (2020). An experimental study on classification of thyroid histopathology images using transfer learning. Pattern Recogn. Lett..

[25] Narayan Das, N., Kumar, N., Kaur, M., Kumar, V., Singh, D. Automated deep transfer learning-based approach for detection of COVID-19 infection in chest X-rays. IRBM [Internet]. 2020 Jul 3 [cited 2021 Nov 30]; Available from: https://www.sciencedirect.com/science/article/pii/S1959031820301172. 10.1016/j.irbm.2020.07.001 PMC7333623 32837679

[26] Albahli S, Albattah W (2020). Detection of coronavirus disease from X-ray images using deep learning and transfer learning algorithms. J. Xray Sci. Technol..

[27] Deng, J., Dong, W., Socher, R., Li, L.-J., Li, K., Fei-Fei, L. ImageNet: A large-scale hierarchical image database. In: 2009 IEEE Conference on Computer Vision and Pattern Recognition. 2009. p. 248–55.

[28] Hernandez-Torres, S., Boice, E.N., Snider, E.J. Development of a tissue phantom for ultrasound imaging and deep learning algorithm training. Ultrasound Med. Biol.

[29] Schindelin J, Rueden CT, Hiner MC, Eliceiri KW (2015). The ImageJ ecosystem: An open platform for biomedical image analysis. Mol. Reprod. Dev..

[30] Schindelin J, Arganda-Carreras I, Frise E, Kaynig V, Longair M, Pietzsch T (2012). Fiji: An open-source platform for biological-image analysis. Nat. Methods..

[31] Image classification | TensorFlow Core [Internet]. [cited 2021 Dec 1]. Available from: https://www.tensorflow.org/tutorials/images/classification.

[32] Zeimarani B, Costa MGF, Nurani NZ, Bianco SR, De Albuquerque Pereira WC, Filho CFFC (2020). Breast lesion classification in ultrasound images using deep convolutional neural network. IEEE Access..

[33] Nwankpa, C., Ijomah, W., Gachagan, A., Marshall, S. Activation functions: Comparison of trends in practice and research for deep learning. arXiv:181103378 [cs] [Internet]. 2018 Nov 8 [cited 2022 Mar 11]; Available from: http://arxiv.org/abs/1811.03378.

[34] Choi, D., Shallue, C.J., Nado, Z., Lee, J., Maddison, C.J., Dahl, G.E. On empirical comparisons of optimizers for deep learning. arXiv:191005446 [cs, stat] [Internet]. 2020 Jun 15 [cited 2022 Mar 11]; Available from: http://arxiv.org/abs/1910.05446.

[35] Yaqub M, Feng J, Zia MS, Arshid K, Jia K, Rehman ZU (2020). State-of-the-Art CNN optimizer for brain tumor segmentation in magnetic resonance images. Brain Sci..

[36] Agnihotri A, Batra N (2020). Exploring Bayesian optimization. Distill..

[37] Frazier, P.I. A Tutorial on Bayesian optimization. arXiv:180702811 [cs, math, stat] [Internet]. 2018 Jul 8 [cited 2021 Dec 1]; Available from: http://arxiv.org/abs/1807.02811.

[38] Sun, L., Xia, C., Yin, W., Liang, T., Yu, P.S., He, L. Mixup-transformer: Dynamic data augmentation for NLP tasks. arXiv:201002394 [cs] [Internet]. 2020 Nov 10 [cited 2022 Mar 11]; Available from: http://arxiv.org/abs/2010.02394.

[39] Zhang, H., Cisse, M., Dauphin, Y.N., Lopez-Paz, D. mixup: Beyond Empirical Risk Minimization. arXiv:171009412 [cs, stat] [Internet]. 2018 Apr 27 [cited 2022 Mar 11]; Available from: http://arxiv.org/abs/1710.09412.

[40] Inoue, H. Data Augmentation by Pairing Samples for Images Classification. arXiv:180102929 [cs, stat] [Internet]. 2018 Apr 11 [cited 2022 Mar 11]; Available from: http://arxiv.org/abs/1801.02929.

[41] Song J, Chai YJ, Masuoka H, Park S-W, Kim S, Choi JY (2019). Ultrasound image analysis using deep learning algorithm for the diagnosis of thyroid nodules. Medicine (Baltimore).

[42] Burgos-Artizzu XP, Coronado-Gutiérrez D, Valenzuela-Alcaraz B, Bonet-Carne E, Eixarch E, Crispi F (2020). Evaluation of deep convolutional neural networks for automatic classification of common maternal fetal ultrasound planes. Sci. Rep..

[43] Liu Y, Wang S, Li Y, Gong Q, Su G, Zhao J (2019). Intraocular foreign bodies: clinical characteristics and prognostic factors influencing visual outcome and globe survival in 373 eyes. J. Ophthalmol..

[44] Snider EJ, Cornell LE, Acevedo JM, Gross B, Edsall PR, Lund BJ (2020). Development and characterization of a benchtop corneal puncture injury model. Sci. Rep..

[45] Snider EJ, Boice EN, Butler JJ, Gross B, Zamora DO (2021). Characterization of an anterior segment organ culture model for open globe injuries. Sci. Rep..

[46] Szegedy, C., Vanhoucke, V., Ioffe, S., Shlens, J., Wojna, Z. Rethinking the Inception Architecture for Computer Vision. arXiv:151200567 [cs] [Internet]. 2015 Dec 11 [cited 2022 Mar 11]; Available from: http://arxiv.org/abs/1512.00567.

[47] Tan, M., Le, Q.V. EfficientNet: Rethinking Model Scaling for Convolutional Neural Networks. arXiv:190511946 [cs, stat] [Internet]. 2020 Sep 11 [cited 2022 Mar 11]; Available from: http://arxiv.org/abs/1905.11946.

