# Supplementary material for: An image classification deep-learning algorithm for shrapnel detection from ultrasound images
Source: Sci Rep. 2022 May 19;12:8427. doi: 10.1038/s41598-022-12367-2 (PMC9117994; doi:10.1038/s41598-022-12367-2)
Supplement: Supplementary file 1 — Supplementary Information. [file 41598_2022_12367_MOESM1_ESM.docx]

## Supplementary Information

Supplementary Script – Python script for the stepwise optimized ultrasound image classification algorithm for the shrapnel detection

**Import Python Libraries**

**import** matplotlib.pyplot **as** plt

**import** numpy **as** np

**import** os

**import** PIL

**import** tensorflow **as** tf

**import** tensorflow.estimator

**import** pathlib

**import** sys

**from** tensorflow **import** keras

**from** tensorflow.keras **import** layers

**from** tensorflow.keras.models **import** Sequential

**from** tensorflow.keras **import** preprocessing

**Import Images from Local Disk for Testing and Validation**

*# Images contain two sub-folders (with and without shrapnel) with near equal image numbers and cropped to remove text. Saved as JPG.*

data_dir **=** pathlib**.**Path(r'E:\CS\TRAIN\Phantom')

image_count **=** len(list(data_dir**.**glob('*/*.jpg')))

positive **=** len(list(data_dir**.**glob('*Shrapnel/*.jpg')))

negative **=** len(list(data_dir**.**glob('*Baseline/*.jpg')))

ratio **=** round(positive**/**negative, 3)

*# Prints the total number of images in directory for clarification, and ratio between file types*

print("Total # of Images = " **+** str(image_count))

print("Shrapnel Images = " **+** str(positive))

print("Baseline Images = " **+** str(negative))

print("Shrapnel/Baseline Ratio = " **+** str(ratio))

**Getting images ready**

*# Resizes images for use with the training, testing, and validating the model*

batch_size **=** 32

img_height **=** 512

img_width **=** 512

input_positive **=** positive**/** 255

input_negative **=** negative**/** 255

class_names **=** ['Baseline', 'Shrapnel']

print(class_names)

*# Build training data set*

train_ds **=** tf**.**keras**.**preprocessing**.**image_dataset_from_directory(

data_dir,

validation_split**=**0.2,

subset**=**"training",

seed**=**123,

image_size**=**(img_height, img_width),

batch_size**=**batch_size)

*# Builds validation data set*

val_ds **=** tf**.**keras**.**preprocessing**.**image_dataset_from_directory(

data_dir,

validation_split**=**0.2,

subset**=**"validation",

seed**=**123,

image_size**=**(img_height, img_width),

batch_size**=**batch_size)

*# Prints random images from the dataset*

plt**.**figure(figsize**=**(10, 10))

**for** images, labels **in** train_ds**.**take(1):

**for** i **in** range(9):

ax **=** plt**.**subplot(3, 3, i **+** 1)

plt**.**imshow(images[i]**.**numpy()**.**astype("uint8"))

plt**.**title(class_names[labels[i]])

plt**.**axis("off")

AUTOTUNE **=** tf**.**data**.**AUTOTUNE

train_ds **=** train_ds**.**cache()**.**shuffle(1000)**.**prefetch(buffer_size**=**AUTOTUNE)

val_ds **=** val_ds**.**cache()**.**prefetch(buffer_size**=**AUTOTUNE)

**Building Model**

num_classes **=** 2 *# number of classes for classification*

model **=** Sequential([

layers**.**experimental**.**preprocessing**.**Rescaling(1.**/**255, input_shape**=**(img_height, img_width, 3)),

layers**.**experimental**.**preprocessing**.**RandomFlip("horizontal", input_shape**=**(img_height,img_width,3)),

layers**.**experimental**.**preprocessing**.**RandomRotation(0.1),

layers**.**experimental**.**preprocessing**.**RandomZoom(0.1),

tf**.**keras**.**layers**.**RandomContrast(0.1),

layers**.**Conv2D(16, 3, padding**=**'same', activation**=**'relu'),

layers**.**MaxPooling2D(),

layers**.**Conv2D(32, 3, padding**=**'same', activation**=**'relu'),

layers**.**MaxPooling2D(),

layers**.**Conv2D(64, 3, padding**=**'same', activation**=**'relu'),

layers**.**MaxPooling2D(),

layers**.**Conv2D(128, 3, padding**=**'same', activation**=**'relu'),

layers**.**MaxPooling2D(),

layers**.**Conv2D(256, 3, padding**=**'same', activation**=**'relu'),

layers**.**MaxPooling2D(),

layers**.**Dropout(0.55),

layers**.**Flatten(),

layers**.**Dense(256, activation**=**'sigmoid'),

layers**.**Dense(num_classes)

], name**=** 'DR_55') *#name outputed in model summary*

model**.**compile(optimizer**=**'RMSprop',

loss**=**tf**.**keras**.**losses**.**SparseCategoricalCrossentropy(from_logits**=True**),

metrics**=**['Accuracy'])

model**.**summary()

callback **=** tf**.**keras**.**callbacks**.**EarlyStopping(monitor**=**'loss', patience**=**3)

epochs **=** 100

history **=** model**.**fit(

train_ds,

validation_data**=**val_ds,

epochs**=**epochs)

*# Save the model*

model**.**save('E:\TrainedModels\DR_55')


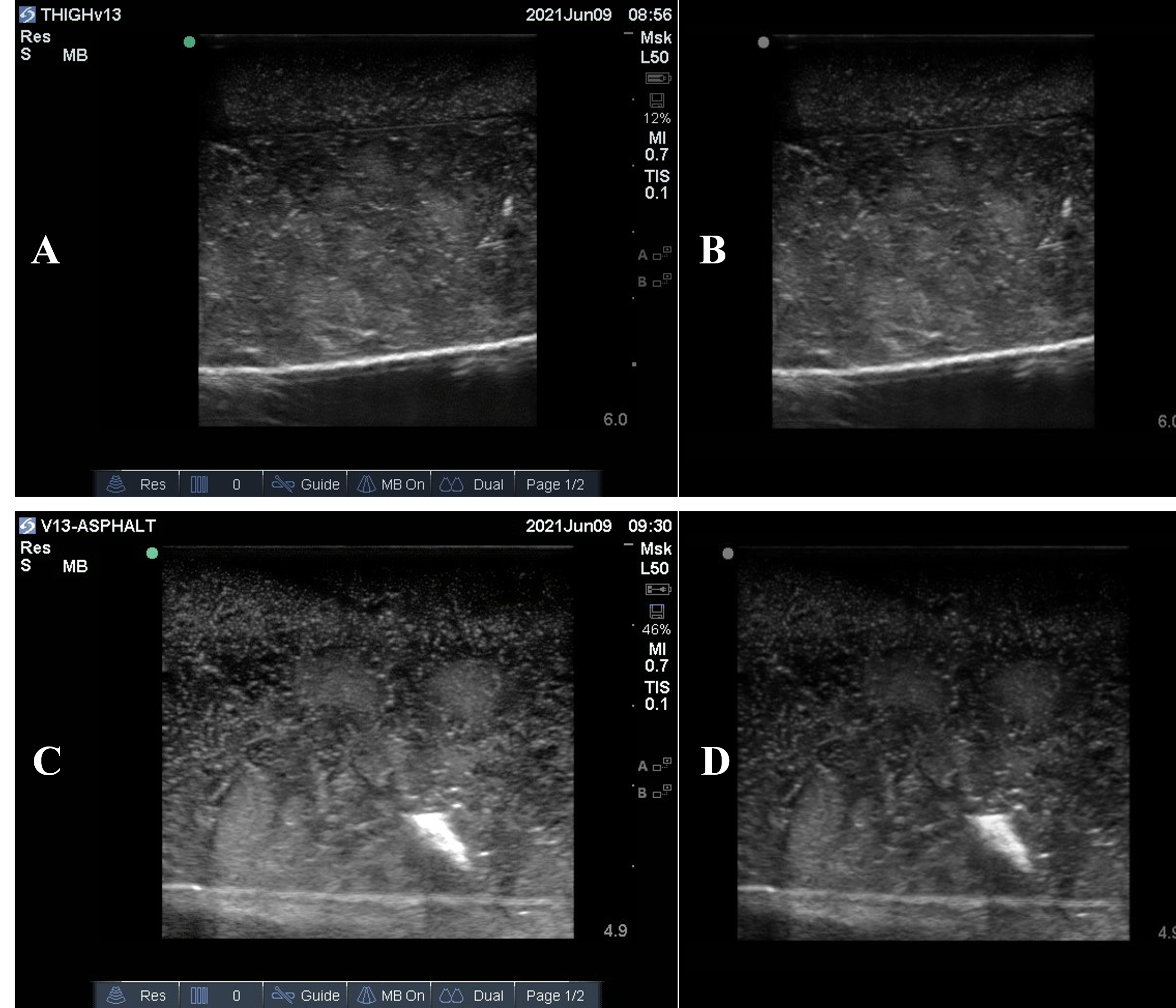


**Supplementary Figure 1. Ultrasound Image Preprocessing**. Representative ultrasound images for preprocessed baseline (**A**), and preprocessed shrapnel (**C**) and postprocessed baseline (**B**) and postprocessed shrapnel (**D**) image types acquired in the gelatin phantom.


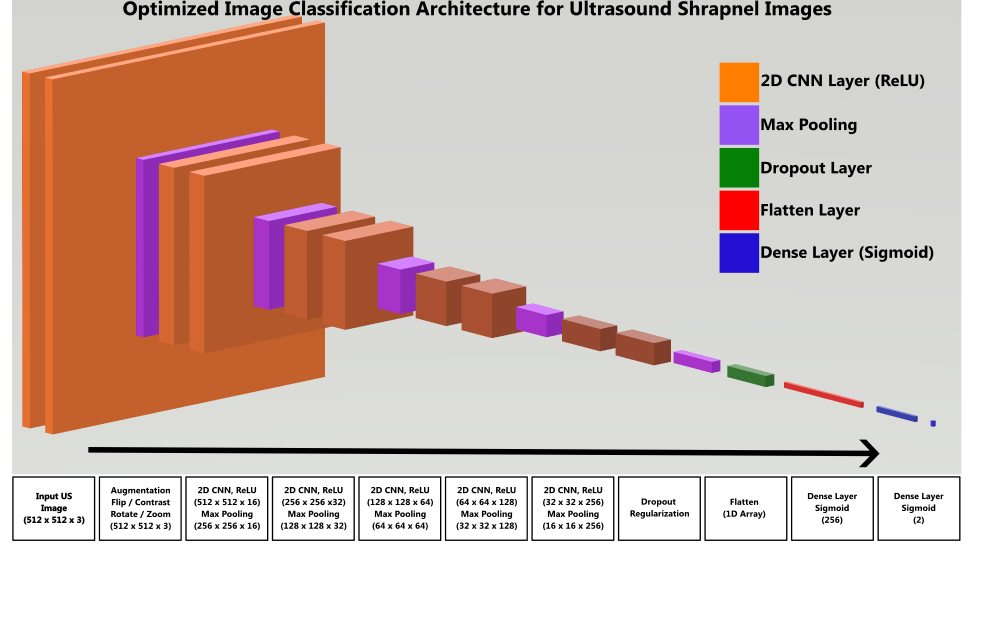


**Supplementary Figure 2. Diagram of the optimized shrapnel classification model.** Architecture diagram for the size and layout for the various layers to the deep learning algorithm after stepwise optimization. A block diagram view showing the image array size changes through the CNN, max pooling, dense, and flatten layers. A more detailed description for the filter sizes and layer architecture is shown in block diagram format at the bottom of the figure.


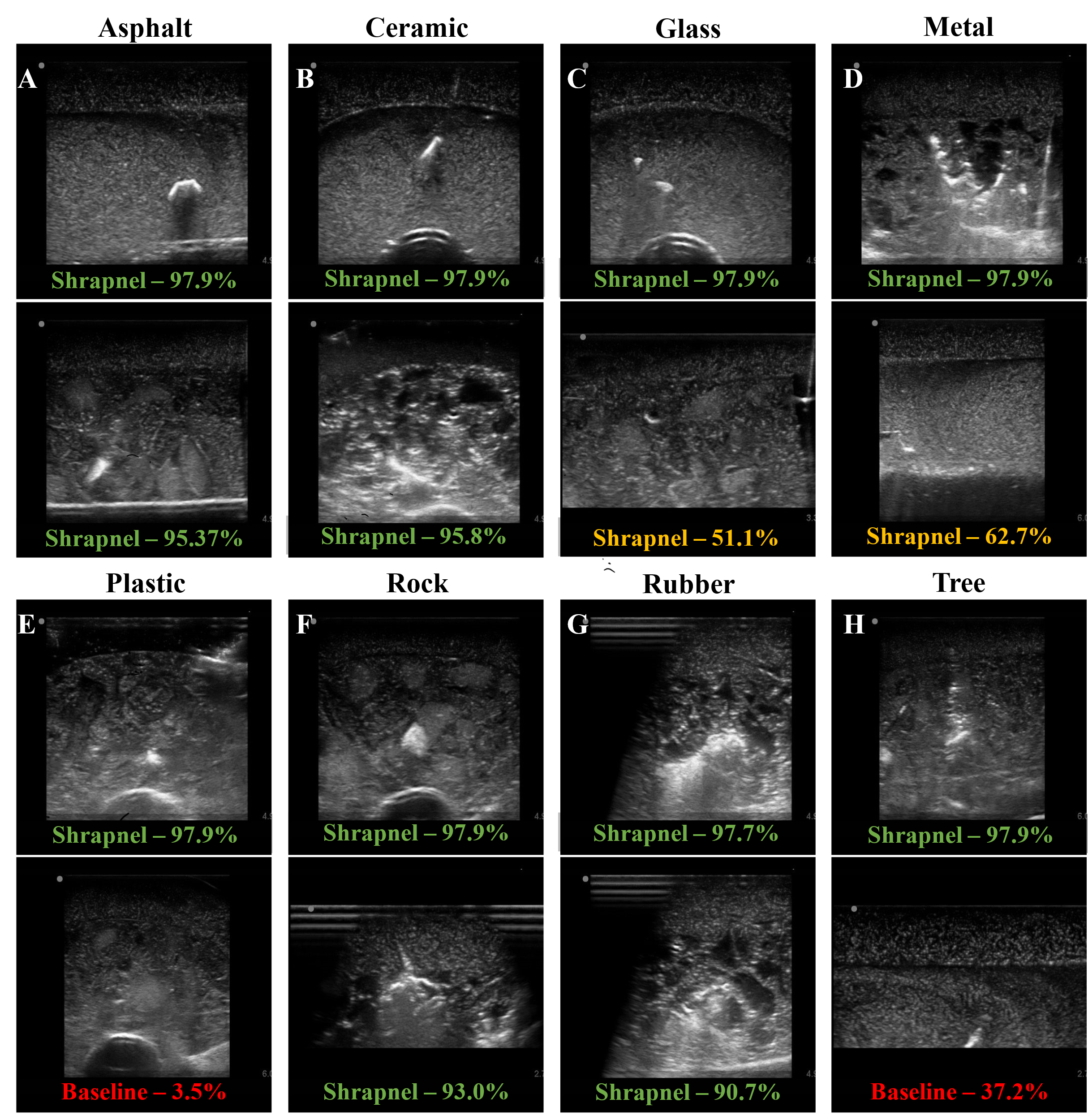


**Supplementary Figure 3. Representative Images for each Shrapnel Type**. Highest and lowest confidence in prediction for every shrapnel type (all images are positive for shrapnel). Shrapnel materials were as follows: asphalt (A, n = 33 images), ceramic (B, n = 28), glass (C, n = 14), metal (D, n = 59), plastic (E, n = 34), rubber (F, n = 10), rock (G, n = 29), and wood (H, n = 23).
